# Studying temporal titre evolution of commercial SARS-CoV-2 assays reveals significant shortcomings of using BAU standardization for comparison

**DOI:** 10.1186/s12985-023-02167-z

**Published:** 2023-09-01

**Authors:** Inge Kroidl, Simon Winter, Raquel Rubio-Acero, Abhishek Bakuli, Christof Geldmacher, Tabea M. Eser, Flora Déak, Sacha Horn, Anna Zielke, Mohamed I. M. Ahmed, Paulina Diepers, Jessica Guggenbühl, Jonathan Frese, Jan Bruger, Kerstin Puchinger, Jakob Reich, Philine Falk, Alisa Markgraf, Heike Fensterseifer, Ivana Paunovic, Angelika Thomschke, Michael Pritsch, Friedrich Riess, Elmar Saathoff, Michael Hoelscher, Laura Olbrich, Noemi Castelletti, Andreas Wieser, KoCo19/ORCHESTRA Study Group, Emad Alamoudi, Jared Anderson, Valeria Baldassarre, Maximilian Baumann, Marc Becker, Franziska Bednarski, Marieke Behlen, Olimbek Bemirayev, Jessica Beyerl, Patrick Bitzer, Rebecca Böhnlein, Isabel Brand, Anna Brauer, Vera Britz, Franziska Bünz, Friedrich Caroli, Josephine Coleman, Lorenzo Contento, Alina Czwienzek, Flora Deák, Maximilian N Diefenbach, Jana Diekmannshemke, Anna Do, Gerhard Dobler, Jürgen Durner, Tabea Eser, Ute Eberle, Judith Eckstein, Manuela Feyereisen, Volker Fingerle, Stefanie Fischer, Felix Forster, Günter Fröschl, Christiane Fuchs, Otto Geisenberger, Mercè Garí, Marius Gasser, Sonja Gauder, Raffaela Geier, Kristina Gillig, Keisha Gezgin, Leonard Gilberg, Kristina Gillig, Philipp Girl, Elias Golschan, Vitus Grauvogl, Jessica Michelle Guggenbuehl Noller, Elena Maria Guglielmini, Pablo Gutierrez, Anslem Haderer, Celina Halfmann, Marlene Hannes, Lena Hartinger, Timm Haselwarter, Jan Hasenauer, Alejandra Hernandez, Luca Heller, Arlett Heiber, Matthias Herrmann, Leah Hillari, Stefan Hillmann, Christian Hinske, Janna Hoefflin, Tim Hofberger, Michael Höfinger, Larissa Hofmann, Kristina Huber, Christian Janke, Lilian Karger, Ursula Kappl, Antonia Keßler, Zohaib Khan, Charlotte Kiani, Isabel Klugherz, Norah Kreider, Johanna Kresin, Arne Kroidl, Pratik Kunder, Magdalena Lang, Clemens Lang, Silvan Lange, Ekaterina Lapteva, Michael Laxy, Ronan Le Gleut, Reiner Leidl, Leopold Liedl, Felix Lindner, Xhovana Lucaj, Elisabeth Lucke, Fabian Luppa, Alexandra Sophie Nafziger, Alexander Maczka, Petra Mang, Paula Matcau, Rebecca Mayrhofer, Anna-Maria Mekota, Dafni Metaxa, Emily Mohr, Hannah Müller, Katharina Müller, Nathalia Nascimento, Kasimir Niermeyer, Sophia Nikolaides, Ivan Noreña, Leonie Pattard, Michael Plank, Claire Pleimelding, Michel Pletschette, Viona Poll, Stephan Prückner, Konstantin Pusl, Peter Pütz, Katja Radon, Elba Raimúndez, Julius Raschka, Christina Reinkemeyer, Camilla Rothe, Viktoria Ruci, Nicole Schäfer, Yannik Schälte, Paul Schandelmaier, Benedikt Schluse, Annika Schneider, Lara Schneider, Sophie Schultz, Mirjam Schunk, Lars Schwettmann, Josefin Sedlmeier, Linda Sintu-Sempta, Alba Soler, Peter Sothmann, Katharina Strobl, Aida Strüber, Laura Strüber, Jeni Tang, Fabian Theis, Verena Thiel, Eva Thumser, Niklas Thur, Sophie Thiesbrummel, Julian Ullrich, Vincent Vollmayr, Emilia Von Lovenberg, Jonathan Von Lovenberg, Carsten Vos, Julia Waibel, Claudia Wallrauch, Nikolas Weigl, Roman Wölfl, Julia Wolff, Pia Wullinger, Tobias Würfel, Patrick Wustrow, Sabine Zange, Eleftheria Zeggini, Thorbjörn Zimmer, Thomas Zimmermann, Lea Zuche

**Affiliations:** 1 Division of Infectious Diseases and Tropical Medicine, Medical Center of the University of Munich (LMU), Munich, Germany; 2 German Center for Infection Research (DZIF), Partner Site Munich, Munich, Germany; 3 Max-von-Pettenkofer Institute, LMU Munich, Munich, Germany; 4 Fraunhofer Institute for Translational Medicine and Pharmacology ITMP, Immunology, Infection and Pandemic Research, Türkenstraße 87, 80799 Munich, Germany; 5 Center for International Health (CIH), University Hospital, LMU Munich, 80336 Munich, Germany; 6 Institute of Radiation Medicine, Helmholtz Zentrum München, 85764 Neuherberg, Germany

**Keywords:** Antibody, COVID-19, Nucleocapsid, RBD, SARS-CoV-2, Serology, Spike, Binding antibody units

## Abstract

**Background:**

Measuring specific anti-SARS-CoV-2 antibodies has become one of the main epidemiological tools to survey the ongoing SARS-CoV-2 pandemic, but also vaccination response. The WHO made available a set of well-characterized samples derived from recovered individuals to allow normalization between different quantitative anti-Spike assays to defined Binding Antibody Units (BAU).

**Methods:**

To assess sero-responses longitudinally, a cohort of ninety-nine SARS-CoV-2 RT-PCR positive subjects was followed up together with forty-five vaccinees without previous infection but with two vaccinations. Sero-responses were evaluated using a total of six different assays: four measuring anti-Spike proteins (converted to BAU), one measuring anti-Nucleocapsid proteins and one SARS-CoV-2 surrogate virus neutralization. Both cohorts were evaluated using the Euroimmun Anti-SARS-CoV-2-ELISA anti-S1 IgG and the Roche Elecsys Anti-SARS-CoV-2 anti-S1 assay.

**Results:**

In SARS-CoV-2-convalesce subjects, the BAU-sero-responses of Euroimmun Anti-SARS-CoV-2-ELISA anti-S1 IgG and Roche Elecsys Anti-SARS-CoV-2 anti-S1 peaked both at 47 (43–51) days, the first assay followed by a slow decay thereafter (> 208 days), while the second assay not presenting any decay within one year. Both assay values in BAUs are only equivalent a few months after infection, elsewhere correction factors up to 10 are necessary. In contrast, in infection-naive vaccinees the assays perform similarly.

**Conclusion:**

The results of our study suggest that the establishment of a protective correlate or vaccination booster recommendation based on different assays, although BAU-standardised, is still challenging. At the moment the characteristics of the available assays used are not related, and the BAU-standardisation is unable to correct for that.

**Supplementary Information:**

The online version contains supplementary material available at 10.1186/s12985-023-02167-z.

## Background

Since the surge of the SARS Corona Virus 2 (SARS-CoV-2) pandemic, considerable progress has been made regarding diagnosis, treatment and prevention of COVID-19. Although by mid-2022, more than 545 million people have been infected and more than 6 million died, serological responses following infection or vaccination are still not fully understood and a correlate of protection has not been identified yet [1, 2].

Describing the natural course of the disease in detail may be key to understanding the immune mechanisms and subsequent protection, either through previous infection or vaccination, or both. Natural infection with SARS-CoV-2 reduces the risk of subsequent infections with the wild-type virus by 82–89% for at least 6 months [3, 4]. In addition, SARS-CoV-2 vaccines protection against symptomatic COVID-19 disease was reported to be 95% for BNT162b2, 94% for mRNA 1273, 70% for ChAdOx1 and 50% for Sinovac [5–9]. The difference in the estimated protective effect of the vaccines correlates with the elicited immune responses, which have been considerably higher in the mRNA vaccines compared to vector-based products [10]. The longevity of this protective effect however, is a matter of debate.

In addition, viral variants of SARS-CoV-2 have emerged since and acquired immune protection was found to be reduced [11]. Large studies have now demonstrated breakthrough infections in vaccinated individuals even during the peak of the antibody response, i.e., weeks or months after completion of the vaccination course [12–15]. A waning of the immune response against SARS CoV-2 was suggested by Mizrahi et al. [14], demonstrating a 1.5 times increased risk for breakthrough infections with the Delta-variant 6 month after vaccination with BioNTech/Pfizer, compared to a 3 month time lag. In an in-house study in early 2022 we observed many breakthrough infections with the Omicron variant regardless of vaccination status or previous infections, including recent infection with the Delta-variant.

Several serological studies have tried to estimate the duration and dynamics of antibody responses following SARS-CoV-2 infection, yielding ambiguous results. Long et al. [16] reported rapid waning of nucleocapsid antibodies in the first 3 months after infection [16, 17]. Similarly, Ibarrondo et al. [18] described a half-life of antibodies against the receptor binding domain (RBD) of 36 days. In contrast, Dan et al. [19], Flehmig et al. [20] and Ripperger et al. [21] reported that immunity against RBD and the anti-Spike domain persisted for at least 7 months. The reasons for these different reports may be e.g. heterogeneity of population, assays used etc.

Several studies highlighted considerable differences in the readout of serological assays, indicating a hampered cross-comparison. In a report by Harris et al. [22], anti-nucleocapsid antibodies measured with the SARS-CoV-2 IgG Assay from Abbott (Abbott Diagnostic, IL, USA), or anti S1 antibodies measured with the Euroimmun Anti-SARS- CoV-2 ELISA IgG (Euroimmun, Lubeck, Germany) were declining within few months. Similarly, plasma from the same subjects measured for anti-nucleocapsid or anti RBD antibodies respectively using the Elecsys Anti-SARS-CoV-2 Roche assays (Roche, Mannheim, Germany), demonstrated stable values over the same time [22, 23].

To improve standardization of serological anti-spike measurements, the WHO made available a set of well-characterized samples deriving from SARS-CoV-2-recovered individuals and shipped by late 2020/early 2021 to laboratories requesting it [24]. These samples were subsequently used to normalize results of different quantitative anti-Spike test systems to standardized units termed “BAU” (Binding Antibody Units) [25]. Many different manufacturers have since published correction factors or formulas to calculate BAU values from their quantitative anti-Spike assays [26]. In addition, laboratories have provided SARS-CoV-2 serology results in BAU to patients and physicians in routine care [27, 28]. Of note, the use of this WHO standard was encouraged to cross validate internal standards, effectively generating a chain of standards [29].

Following the suggested approach, we compared anti-spike antibody titres quantitatively at defined and standardized time points spanning over 18 months after infection using different commercially available test kits. Therefore, we used samples derived from ninety-nine SARS-CoV-2-infected individuals and from forty-five participants with no history of previous infection but with two vaccinations. The anti-spike quantitative responses were calculated to BAU units as suggested by the manufacturers and compared. Two assays reacting only to infection were added to the analysis.

## Methods

### Cohort members: patients and vaccinees

**Fig. 1:**
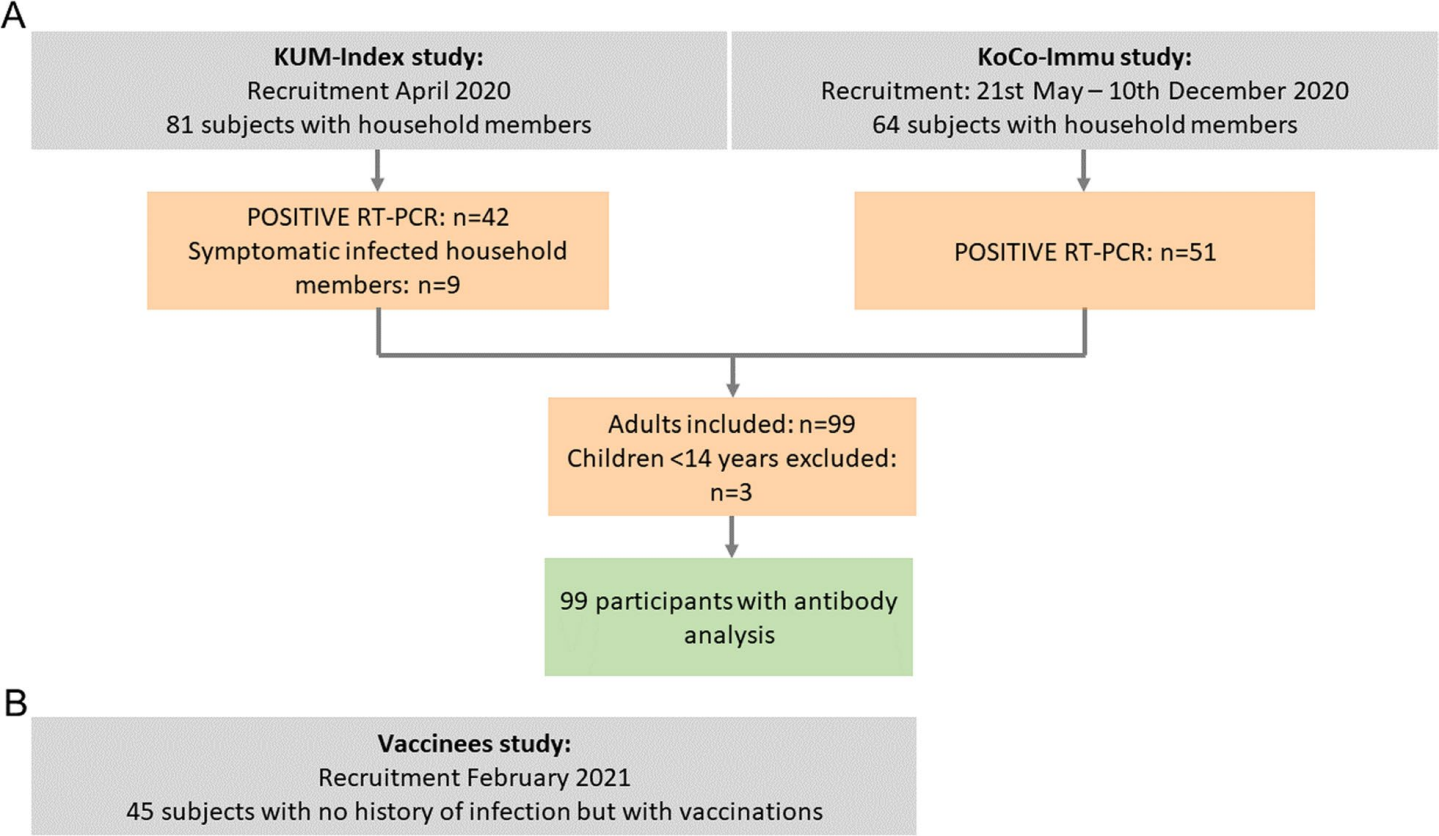
Cohort flow chart. **A** Cohort of non-vaccinated SARS-CoV-2 RT-PCR positive subjects. Two recruitment strategies were used: fifty-one participants, who had a SARS CoV-2 infection in February/March 2020 were recruited in April 2020 together with their household members (KUM-Index-study). Forty-two of them had a positive PCR and additional 9 household members developed SARS CoV-2 specific antibodies. From 21 May till 10 December 2020 another fifty-one SARS-CoV-2 infected individuals were recruited as early as possible after their first positive RT-PCR (Koco19-Immu-Study). **B** Cohort of vaccinees. Forty-five participants with no history of infection but with two vaccinations were recruited

From April to December 2020, 66 households were included in the study with all household members, irrespective of a SARS-CoV-2 infection (Fig. 1A). A total of 145 non-vaccinated participants were enrolled, including 102 members infected with SARS CoV-2. For the three children below the age of 14, no venous blood draw was performed, for the remaining ninety-nine patients venous blood was drawn as soon as possible after the first positive RT-PCR and at multiple time points thereafter.

Additionally, it was possible to recruit forty-five participants with no history of previous infection but with two vaccinations (Fig. 1B). A possible previous infection was excluded with all the following criteria: (i) no past positive RT-PCR for SARS-CoV-2, (ii) past COVID-19 like symptoms had to be followed by a negative RT-PCR, (iii) negative serology deriving from infection (Roche Elecsys Anti-SARS-CoV-2 anti-N, see next paragraph for details), and (iv) negative SARS-CoV-2 surrogate virus neutralization test (GenScript®, see next paragraph for details). The latest performed only at recruitment, end of follow-up and at sporadic time-points.

Sample collection was performed as previously presented [30].

On December 1st 2020, this cohort joined the ORCHESTRA (Connecting European Cohorts to Increase Common and Effective Response to SARS-CoV-2 Pandemic) project but was not previously published.

### Laboratory analysis

Serologic assays were performed using EDTA-plasma samples and were conducted as previously published [23, 30, 31]. The serological assays used were chosen if: available in large quantities, performable with semi-automated workup, acceptable pricing, licensed for the use in Europe, and well-described in performance [23]. The manufacturer’s instructions were followed for all assays. For sample time-points of PCR-positive participants the following assays were performed: Euroimmun Anti-SARS-CoV-2-ELISA anti-S1 IgA/IgG (hereafter called EI-S1-IgA/EI-S1-IgG; Euroimmun, Lübeck, Germany), Quantitative Euroimmun Anti-SARS-CoV-2-QuantiVac ELISA (IgG) (hereafter called EI-S1-IgG-quant; Euroimmun, Lübeck, Germany), Roche Elecsys Anti-SARS-CoV-2 anti-N and Elecsys Anti-SARS-CoV-2 S anti-S1 (hereafter called Ro-N-Ig and Ro-RBD-Ig-quant, respectively; Roche, Mannheim, Germany) and GenScript® (hereafter called GS-cPass, Piscataway, New Jersey, USA). For sample time-points of vaccinees two assays were performed: Ro-RBD-Ig-quant and Quantitative Euroimmun Anti-SARS-CoV-2-QuantiVac ELISA (IgG) (hereafter called EI-S1-IgG-quant; Euroimmun, Lübeck, Germany). Values of EI-S1-IgG-quant and Ro-RBD-Ig-quant could be obtained in BAUs.

Multiple measurements of the same sample (operational replicates) were performed on different days with different operators and lots to control the intra-variability of all the assays. The very good intra-variability of EI-S1-IgG and Ro-N-Ig was already published [23]. For GS-cPass and Ro-RBD-Ig-quant evaluation was performed with in house samples and similar results as for the published assays were obtained (data now shown here).The World Health Organization (WHO) reference sera (National Institute for Biological Standards and Control [NIBSC] code 20/268) were measured on the assays EI-S1-IgG, EI-S1-IgG-quant, Ro-N-Ig and Ro-RBD-Ig-quant in replicates (n = 3) to standardize the results [31]. In this analysis we present only the mean value.

### Data analysis

Prior to analysis, the data was cleaned and locked, so that no new measurements can be included after review. For operational replicates, the first measurement of EI-S1-IgG was used, since small losses compared to fresh samples were found. In the case of Ro-N-Ig and GS-cPass the latest measurement was included, while for Ro-RBD-Ig-quant the most diluted value still within the linear range was selected to calculate the true unit count. The software R, 4.0.5 (https://cloud.r-project.org/) was used to perform statistical analysis and visualisation. Longitudinal serological dynamics were analysed applying the LOESS (locally estimated scatterplot smoothing or local regression) method with the 95% CI.

## Results

### Cohort description

After recruitment of 190 individuals, a total of 144 participants were included in the analysis, 69% (99/144) of which were oligo-symptomatic non-vaccinated SARS-CoV-2 RT-PCR positives and the remaining were subjects with no history of previous infection but with two vaccinations (hereafter called vaccinees).

For the non-vaccinated RT-PCR positive individuals a total of 438 study visits were conducted, between 1 and 8 per participant. The median age at enrollment was 37.8 years; 61% (60/99) of the participants were females. For the vaccinees (29/45, 64.4% females) 250 blood samples were collected, 92 (36.8%) before and 158 (63.2%) after the second vaccination. The time of collection after the second vaccination varied between 1 and 236 days (mean = 43.57 days and median = 6 days). In the first vaccination the vaccines used were Biontech Pfizer (27/45, 60.0%), AstraZeneca (11/45, 24.4%) and Johnson & Johnson (6/45, 13.3%), while for the second vaccine dose, it changed to Biontech Pfizer (37/45, 82.2%), Moderna (5/45, 11.1%) and AstraZeneca (1/45, 2.2%).

### Premedical history and symptoms during infection

A small percentage of the non-vaccinated PCR-positive participants reported chronic medical conditions (diabetes mellitus, heart disease or hypertension, 13%) and an additional 5% reported known allergies. During the initial phase of the SARS CoV-2 infection, symptoms were recorded and classified according to the WHO classification (Additional file 1: Fig. S1) [29, 30]. In total, 7% (7/99) of the participants were classified as WHO-category 1, 44% (43/99) as WHO-category 2 and 48% (47/99) as WHO-category 3. One third (14/43) of the WHO-category 2 patients reported clinically significant involvement of the lower respiratory tract, while for WHO-category 3 patients the proportion rose to two thirds (30/47). Additionally, two participants had to be hospitalized due to the severity of the symptoms. For further analysis, we divided the participants in two groups, WHO 1–2 and WHO ≥ 3. Analysis of WHO-classification can be found in the Additional file 1.

### Sero-positivity at baseline measurements after SARS-CoV-2 infection

**Fig. 2:**
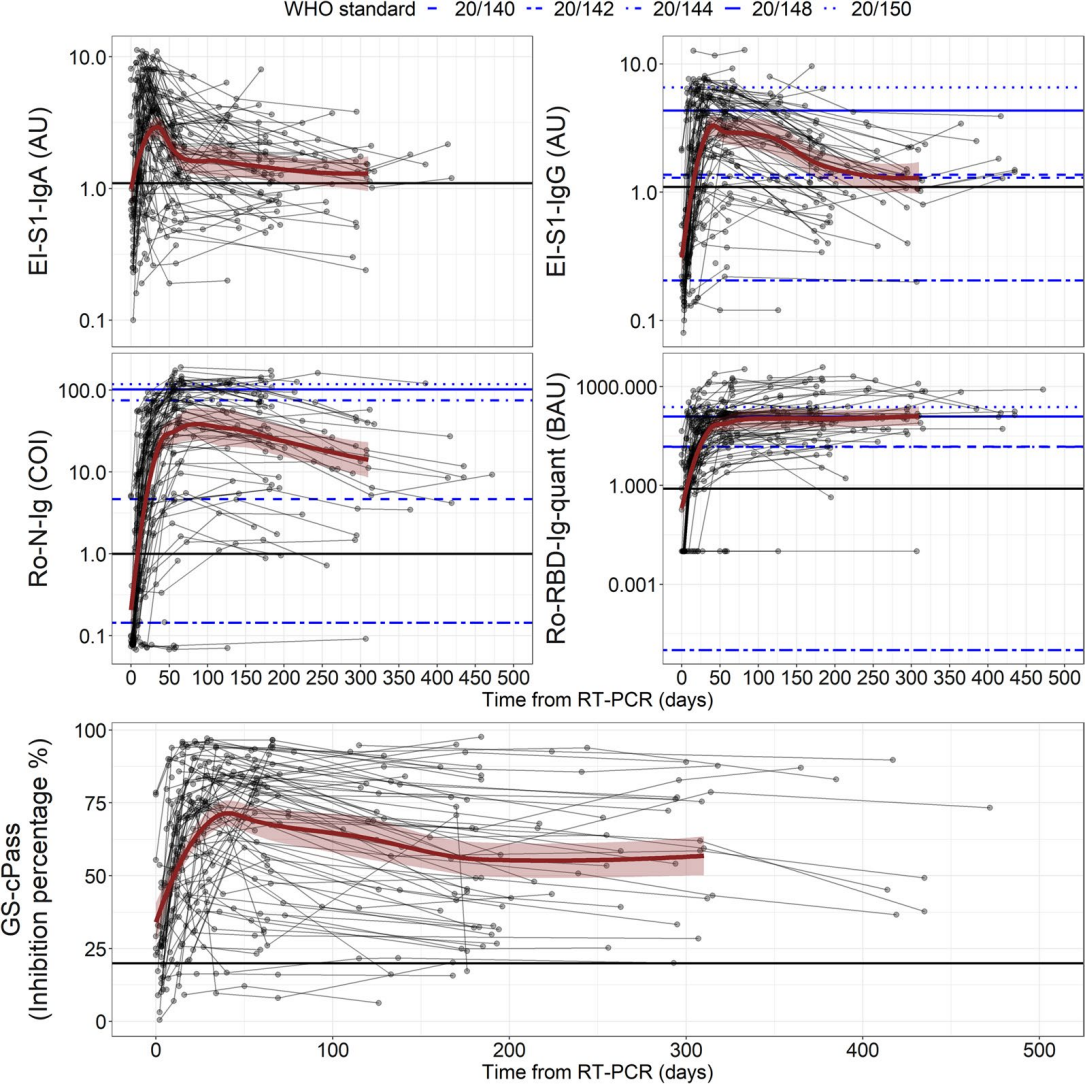
Longitudinal serological dynamics of SARS-CoV-2 RT-PCR positive cohort. Solid black horizontals line denote the cut-off for positivity. Blue lines represent the WHO reference panel for anti-SARS-CoV-2 immunoglobulin (NIBSC code 20/268). Each line represents one subject, the dots represent the individual samples. All assays were performed from the same sample in a head-to-head comparison. Top left: Euroimmun Anti Spike IgA; top right: Euroimmun Anti-Spike IgG; middle left: Roche Anti-Nucleocapsid; middle right: Roche Anti Spike/RBD; bottom: GenScript neutralization surrogate test

The longitudinal serological dynamics of non-vaccinated SARS-CoV-2 RT-PCR positive individuals was followed using five assays for head-to-head comparison (Fig. 2). The baseline measurements yielded positive sero-responses in 57% (56/99) of the samples for EI-S1-IgA, 44% (43/99) for EI-S1-IgG, 33% (32/99) for Ro-RBD-Ig-quant, 53% (52/99) for Ro-N-Ig, and 83% (81/99) for GS-cPass. 31% (30/99) of the cohort was seroconverted in all assays, while negative results in all assays were recorded in 16% (16/99). Over time, 79% (78/99) of participants seroconverted as detected by all assays while 9% (9/99) did not develop any or solely very low antibody titres (GS-cPass or GS-cPass and Ro-N-Ig/EI-S1-IgA/G positive). For three subjects, a potentially false positive RT-PCR test result was discussed due to complete lack of any clinical symptoms or other signs for SARS-CoV-2 infection. The remaining 12% (12/99) of participants had a measurable sero-response in at least three of the five assays used.

Time to seroconversion was determined in those participants with initial negative readouts. Here, mean EI-S1-IgA positivity was detected 20 days (min = 8 days, max = 69 days) after symptom onset, EI-S1-IgG positivity after 31 days (min = 14 days, max = 118 days), Ro-RBD-Ig-quant positivity after 20 days (min = 7 days, max = 133 days), positive Ro-N-Ig reaction after 30 days (min = 10 days, max = 118 days), and GS-cPass-positivity after 16 days (min = 11 days, max = 31 days).

### Longitudinal serological dynamics after SARS-CoV-2 infection

Subsequently, we compared the quantitative reactivity of the test systems over time (Fig. 2). For the anti-S1/anti-RBD tests, the EI-S1-IgA peaked fastest (35, 31–39 days) and declined rapidly at first, followed by a phase of slower decay at > 86 days. Using the same antigen but measuring IgG, we saw the peak/slope change much later (47, 43–51 days) and a subsequent slower decay (> 208 days). Values obtained by the Ro-RBD-Ig-quant test rose similarly fast (47, 43–51 days), however, without any decline over time as observed in both EI-S1-IgG and IgA assays. Comparing the results with GS-cPass we observed an initial peak reached after 43 (39–47) days which was similar to the dynamics measured by EI-S1-IgG and Ro-RBD-Ig-quant. Afterwards, the inhibition declines and plateaus at a level of about 55.5% (55–56%). The sero-response of the Ro-N-Ig assay peaks later (75, 71–78 days) compared to EI-S1-IgG and subsequently declined almost linearly (122, 118–126 days).

**Fig. 3:**
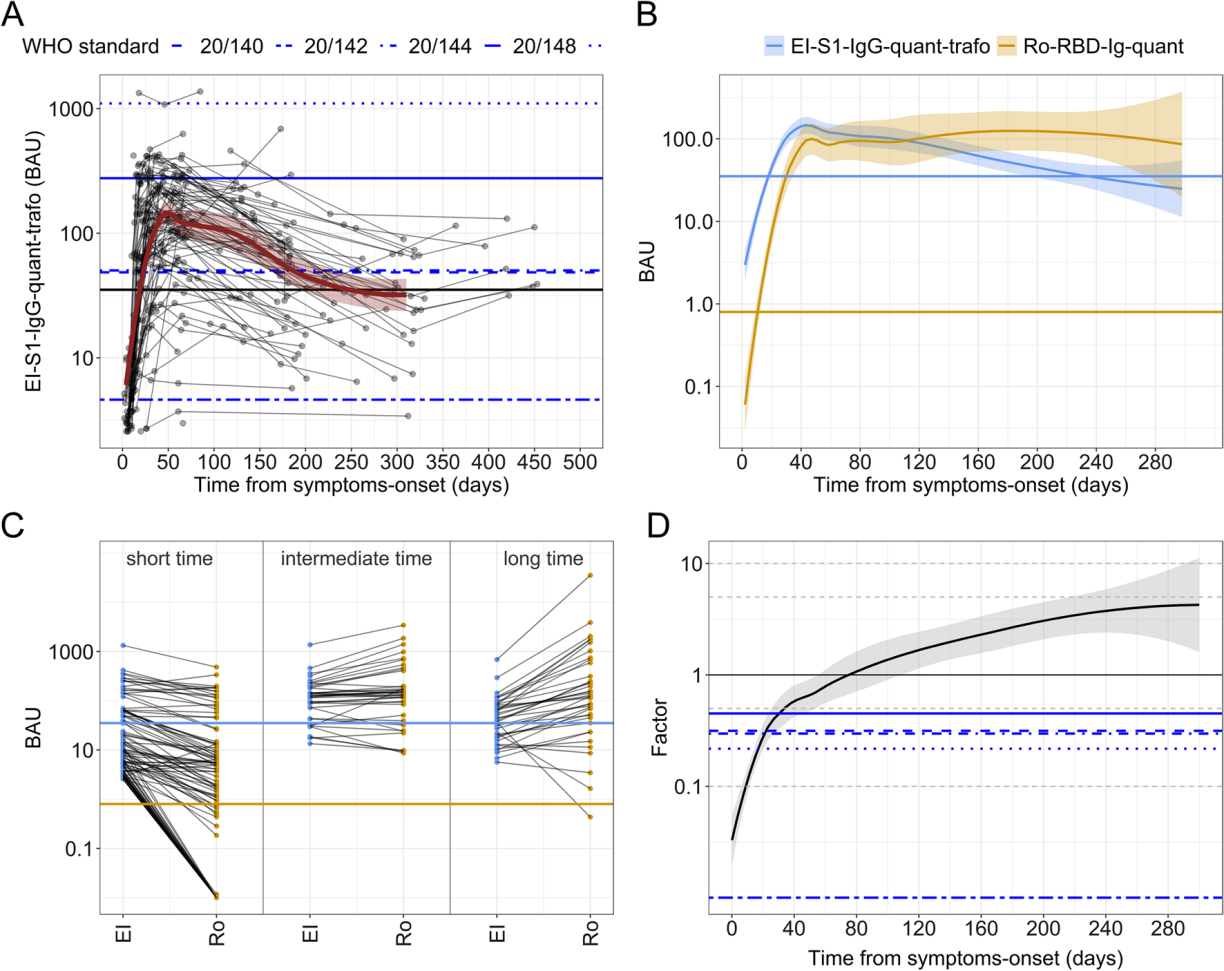
Comparison of quantitative serology of individual patients (PCR-positive cohort) over time for EI-S1-IgG-quant-trafo and Ro-RBD-Ig-quant. When assays are compared, the EI-S1-IgG-quant-trafo is represented in blue while Ro-RBD-Ig-quant in yellow. Blue lines represent the WHO reference panel for anti-SARS-CoV-2 immunoglobulin (NIBSC code 20/268). Solid horizontal lines represent the cut-off for positivity. **A** Longitudinal EI-S1-IgG-quant-trafo serology data over time. Each line represents one subject, the dots represent the individual samples. The red solid line shows the LOESS (locally estimated scatterplot smoothing or local regression) estimations with CI in translucent red. **B** Aggregated BAU value curves of the subjects over time for the two tests. EI-S1-IgG-quant-trafo rises faster, reaches similar values than Ro-RBD-Ig-quant between days 70 and 150 after infection and then drops to about 1/10th of the value observed in Ro-RBD-Ig-quant after one year (please note that here almost half of the samples measured in EI-S1-IgG-quant-trafo are already below the positivity threshold). **C** Parallel coordinate plot dividing the time from symptom onset into three intervals: short (0–20 days), intermediate (70–150 days) and long (170–250 days). EI denotes the EI-S1-IgG-quant-trafo assay while Ro represents the Ro-RBD-Ig-quant assay. **D** The Quotient of the two BAU values depicted in log10 scale over time (Ro-RBD-Ig-quant/EI-S1-IgG-quant-trafo). At the Factor Value of 1, the BAU values are identical. This occurs only in a short timeframe about 80 days post symptom onset

In a second step, we aimed to compare the non-quantitative readouts of EI-S1-Ig with the quantitative readouts of Ro-RBD-Ig-quant. For this purpose non-quantitative EI-S1-Ig values were transformed into quantitative EI-S1-Ig-quant (called EI-S1-Ig-quant-trafo). Details on the procedure are outlined in the Additional file 1 and the longitudinal analysis is presented in Fig. 3A. After standardization for BAU, the paired values of EI-S1-IgG-quant-trafo and Ro-RBD-Ig-quant were compared (Fig. 3B). The EI-S1-IgG-quant-trafo peaked at day 43 (40–47) with a mean value of 147.04 (116.88–184.98) BAU, while Ro-RBD-Ig-quant reached its maximum at day 47 (43–51) with a mean value of 100.20 (65.09–154.26) BAU.

### EI-S1-IgG-quant-trafo and Ro-RBD-Ig-quant assays after SARS-CoV-2 infection

In order to examine in more depth the differences between the assays EI-S1-IgG-quant-trafo and Ro-RBD-Ig-quant, three time bands for time since symptom onset were defined: (i) short time (0–20 days, increase phase of antibody titres), (ii) intermediate time (70–150 days, plateaued antibody titers) and (iii) long time (170–250 days, decrease phase of antibody titres). Time intervals are not equally long and there are gaps in the timeline to better define the three phases of the serological dynamic.

The parallel coordinate plot (Fig. 3C) demonstrates that the two assays yielded differing results and were only similar in the intermediate time band. Subsequently, the quotient between the measured BAU values was calculated in an effort to quantify the differences observed, whereby a factor of 1 implied the same readout in both tests. This, however, was only observed at day 80 after symptom onset with values differing greatly before and after (Fig. 3D). Shortly after infection, multiplication by factor 0.1 was necessary to obtain similar values of EI-S1-IgG-quant-trafo compared to Ro-RBD-Ig-quant, whereas after 250 days the factor was 5. In addition, the differences are likely to be underestimated, as a correction was no longer possible if one test dropped below detection limit. This occurred in almost half (48.65%; 18/37) of the EI-S1-IgG-quant-trafo values and only in less than 5% (2/41) in the Ro-RBD-Ig-quant at the 250 day mark. The WHO reference panel for anti-SARS-CoV-2 immunoglobulin (NIBSC code 20/268) resulted to have higher values in EI-S1-IgG-quant-trafo compared to Ro-RBD-Ig-quant in all samples (Fig. 3D, quotient smaller than 1).

### EI-S1-IgG-quant-trafo and Ro-RBD-Ig-quant assays after twice vaccination against SARS-CoV-2

**Fig. 4:**
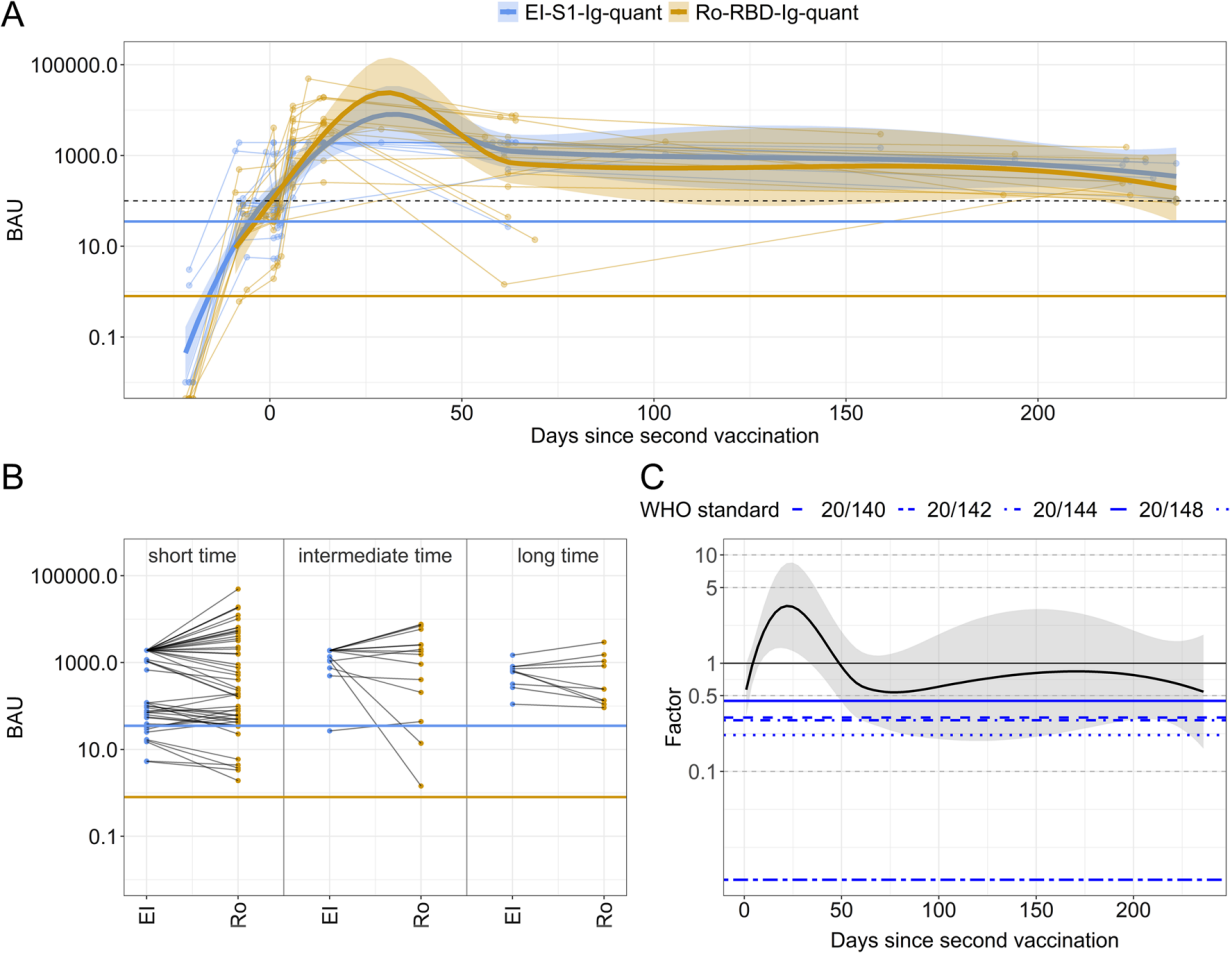
Comparison of individual Anti-S1 BAU values of vaccinees over time for EI-S1-IgG-quant and Ro-RBD-Ig-quant, respectively. The time zero denotes the day of the second vaccination. The EI-S1-IgG-quant is represented in blue while Ro-RBD-Ig-quant in yellow. Solid horizontal lines represent the cut-off for positivity. **A** Longitudinal serology data of subjects over time. Each line represents one subject, the dots represent the individual samples. The solid lines show the LOESSs (locally estimated scatterplot smoothing or local regressions) estimations with CI as a shadowed region. The dashed black lines denotes 250 BUS/mL. **B** Parallel coordinate plot dividing the time from symptom onset into three intervals: short (0–20 days), intermediate (70–150 days) and long (170–250 days). EI denotes the EI-S1-IgG-quant assay while RO the Ro-RBD-Ig-quant assay. **C** Quotient of the two BAU values depicted in log10 scale over time (Ro-RBD-Ig-quant/EI-S1-IgG-quant-trafo); at 1, the BAU values are identical. Blue lines represent the WHO reference panel for anti-SARS-CoV-2 immunoglobulin (NIBSC code 20/268)

Serological dynamics, assay readouts, and BAU values from the non-vaccinated SARS-CoV-2 infected cohort were compared to healthy controls vaccinated twice against SARS-CoV-2. Therefore, EI-S1-IgG-quant and Ro-RBD-Ig-quant assays were measured in blood samples before and after second vaccination (Fig. 4A). Serological dynamics and temporal evolution of the titers measured by EI-S1-IgG-quant and Ro-RBD-Ig-quant differed considerably in naturally infected individuals, this was not the case for vaccinated individuals. As Fig. 4B and C demonstrate, parallel coordinate plots were less divergent and the ratio of the two assays showed to be closer to 1. After a first increase phase (from day 0 to day 34 (30–37) for EI-S1-IgG-quant and to day 31 (28–34) for Ro-RBD-Ig-quant), the median peak was reached with a level of 8069.21 (1912.37–34,047.96) BAU for EI-S1-IgG-quant and 23,988.33 (4073.80–144,543.98) BAU for Ro-RBD-Ig-quant. Both serological dynamics then decrease rapidly until day 63 (60–66) or 62 (59–65) for EI-S1-IgG-quant or Ro-RBD-Ig-quant, respectively. Thereafter, the slopes of the decrease reduce greatly.

### Natural infection versus double SARS-CoV-2 vaccination

In our study, antibody-titers in fully vaccinated (i.e., twice) non-infected individuals were considerably higher compared to the naturally infected participants. For EI-S1-IgG-quant, the difference between the maximum value for vaccinated vs. naturally infected was 7.92217 BAU, while for Ro-RBD-Ig-quant the difference increased to 13.96833. In total, 93.75% of the vaccinees reached a maximum value over 1000 BAU, while only 69.7% of the vaccination naive infected subjects reached a value over 100 BAU.

In contrast to natural infections, vaccinated individuals exhibited a sharp decline in antibody titers as determined by Ro-RBD-Ig-quant, limited to approximately 60 days after the second vaccination followed by a plateau of titer (Fig. 3B compared to Fig. 4A). EI-S1-IgG-quant presented a similar profile, but in this assay a sharp decrease was also observed after natural infection. After vaccination however, the observed velocity of decrease seems to reduce. In mean, both assays yield positive readouts over the period analyzed, with values of the EI-S1-IgG-quant assay being closer to the positivity threshold compared to the ones as determined by Ro-RBD-Ig-quant.

## Discussion

In this study, we compared serological dynamics using samples from ninety-nine non-vaccinated PCR-positive participants and from forty-five participants with no history of previous infection but with two vaccinations against SARS-CoV-2. Serum samples were analyzed with a total of six different assays. To follow infection, assay readouts of Ro-N-Ig and GS-cPass were performed from the same sample in a head-to-head comparison. Participants showed positive antibodies against these assays for at least 400 days. The remaining four assays detect responses to both infection and vaccination. In previous studies the EI-S1-IgA showed to be less reliable [23] and was therefore performed only for samples of the PCR-positive participants. The EI-S1-IgG assay is per manufacturer’s definition non-quantitative and its quantitative version is defined by the EI-S1-IgG-quant assay [31]. It was possible to measure samples of the PCR-positive participants only with the non-quantitative version. Measurements were hence transformed to quantitative values using paired samples presented in [31]. A comparison with the vaccinees was possible thereafter, together with direct readouts of the Ro-RBD-Ig-quant for both cohorts. As a results, the longitudinal dynamics of EI-S1-IgG-quant and Ro-RBD-Ig-quant in the PCR-positive cohort present completely different trends, while for vaccinees the trends a very similar.

Duration and magnitude of serological responses in relation to different testing systems and antigen-target has been subject to dissent. Harris et al. [22] demonstrated a rapid decline of anti-N antibodies using the ELISA from Abbot, with only 51% of SARS-CoV-2 infected individuals having a sero-response after 6 months. In contrast, Favresse et al. [32] showed a positivity rate of 94% after 10 months but using the Ro-N-Ig test. Muecksch et al. [33] compared four different assays: the Ro-N-Ig and the Abbott SARS-CoV-2 immunoglobulin (Ig) G assay for anti-N detection, and the DiaSorin SARS-CoV-2 IgG together with the Siemens SARS-CoV-2 RBD assay for anti-S comparisons. Similarly to our analysis, the shapes of the curves strongly differ between assays. Dan et al. [19] described a half-life of binding anti-S antibodies of 103 days. In contrast Ibarrondo et al. [18] described a rapid decay of anti-RBD antibodies with a half-life of only 36 days.

In our cohort of non-vaccinated SARS-CoV-2 RT-PCR-positive individuals direct readout values are coherent to previous published literature, comparing the same testing platform [33]. If compared to other assays, same discrepancies as in the rest of the literature appear. In addition, clinical characteristics of the underlying cohorts differed greatly. Several authors described a correlation between magnitude of antibody responses and degree of clinical symptoms in SARS-CoV-2 infected individuals [16, 17, 32, 34–38]. This was replicated in our cohort, where we could observe a trend towards higher antibodies titers in individuals with more severe symptoms. As we solely enrolled oligo-symptomatic participants, these findings did not reach statistical significance. Of importance, exactly that group of oligo-symptomatic patients is the overwhelming majority of the population which might be subject to serological testing for different reasons.

A correlate of protection of SARS-CoV-2 has not been established yet and it is still debated whether the protection after natural infection is different or perhaps even superior to vaccination [39]. Natural infection is likely to elicit a broader response against more epitopes of the virus [40]. However, several studies describe the immune response after vaccination to be characterized by higher antibody levels compared to natural infections, especially following vaccination with mRNA- based vaccines [5, 7, 9, 33, 41]. Recent reports describe waning protection already shortly after the second dose and the decay seems more pronounced than after a natural infection [42–45]. Similarly, when comparing naturally infected to vaccinated participants, we observed pronouncedly higher antibody levels in the latter compared to the former. Antibody levels remained positive for at least seven months after vaccination.

Initial reports on SARS-CoV-2 infected cohorts declared a high level of protection of 82–89% for approximately 6 months against the wild type virus [3]. Similarly, data from Israel suggested a high level of protection after vaccinating with Pfizer-BioNTech [46]. Since the surge of new virus variants protection against Delta and Omicron variants was reduced [14]. Mizrahi et al. [14] described a 1.5 times increased risk for breakthrough infections with Delta variant for subjects 6 months after vaccination with Pfizer-BioNTech compared to subjects 3 months after vaccination. Shrotri et al. [42] compared protection of vaccinated individuals with anti-RBD antibodies above and below 500 BAU (ELYSYS Ro-Ig) and found significantly more infected participants with antibodies below 500 BAU. However, our data suggests that an antibody lever of 500 BAU is usually not reached after natural infection. Our SARS-CoV-2 RT-PCR-positive cohort only included participants infected with the original wild type strain. A comparison between variants is therefore not possible, but would also only generate data unclear to compare, as the vaccines and the antigens used in the tests are all also wild type.

Modelling the temporal evolution of the antibody levels, the serological dynamics of the vaccinated cohort is completely different than that of the PCR-positive vaccine naive infected participants. After vaccination, we observed a short initial peak phase, followed by a very slow decline of antibody levels in both quantitative tests EI-S1-Ig-quant and Ro-RBD-Ig. All measurements were above the positivity threshold even seven months after the second dose. The curves representing the antibody dynamics of both quantitative tests were very similar. This is in contrast to our observations in the SARS-CoV-2 RT-PCR-positive cohort described here. The ELISA-based Euroimmun test suggested a rapid decline of antibodies with more than 50% of the samples dropping below the threshold for positivity within less than one year while the Ro-RBD-Ig assay yielded positive readouts after 200 days almost without declining. An explanation for the slow EI-S1-Ig-quant antibody decrease in vaccinated versus the steady state suggested by Ro-RBD-Ig readout in the RT-PCR-positive cohort could be the rise in avidity as also described by Scheiblauer et al. [47]. The authors hypothesized that two vaccine doses lead to an antibody response dominated by highly specific and highly avid IgG directed against the S-protein. Thus, the antibody-signal dynamics over time reflect the overall amount of antibodies in both tests. In contrast, natural infection will likely elicit a much broader response which maturates over time [47], including detection of the RBD-domain which in turn might lead to an increase in avidity, while the overall antibody amount is dropping [47]. Those two opposing effects may compensate each other at different rates depending on the assay format. The Ro-RBD-Ig assay reportedly detects the binding of few antibodies but favors high avidity [47], potentially resulting in a persistently high assay readout, while the ELISA-based Euroimmun assay values are biased towards whole antibody binding and thus decline. Persisting non-declining Roche RBD-antibodies detectable for more than 300 days after natural infection have been repetitively described with a level of ~ 100 BAU [32, 47].

Summarising, we present the results of a well-characterized cohort to investigate dynamics in serological responses to non-vaccinated SARS-CoV-2 infected individuals compared to vaccinated healthy controls. For quantitative anti-Spike assays, we used BAU standardization which is provided by the manufacturer.

However, we observe distinct differences both in the magnitude and dynamics of the measured antibody response, although BAU standardization for anti-S1/RBD tests was used. Interestingly, these differences were negligible for samples taken two months after symptom onset. The standardization however is less accurate before and after this time period, resulting in differences of up to one order of magnitude in supposedly standardized and comparable values. These differences disappeared in the vaccinated cohort. One potential explanation could be the fact that the assays measure different targets. While the EI-S1-IgG detects the overall amount of binding antibodies in an ELISA-format, Ro-RBD-Ig-quant is an ELECSYS based double-antigen sandwich-test, detecting highly avid antibodies [47]. Importantly, the BAU-standard provided by the WHO is derived from a group of donors relatively shortly after the infection. Subsequently, standardization performed for an assay will likely be accurate for tests with a similar profile of antibodies, regarding both subclass as well as avidity, hence only in individuals few months after natural infection [25]. Therefore, it is not be the best standard for clinical cohorts, including samples from individuals very early or late after the infection, or after vaccination.

## Conclusion

The data presented here suggest that the establishment of a protective correlate or vaccination booster recommendation based on BAU might be hampered. Also, comparisons of individual patient values between different laboratories will be unreliable even if reported in BAU. The characteristics of the individual test systems employed need to be considered and should be corrected for, as the differences are likely to be high especially in subjects with small amounts of highly avid antibodies.

### Supplementary Information

**Additional file 1: Figure S1** WHO grading of symptoms (from [29]). **Figure S2** Longitudinal serology data of patients (PCR-positive cohort) over time colored by WHO symptom grade. Each line represents one subject, the dots represent the individual sample. All assays were performed from the same sample in a head-to-head comparison. The blue and red solid lines show the LOESS (locally estimated scatterplot smoothing or local regression) estimations with CI. Top left: Euroimmun Anti Spike IgA; Top right: Euroimmun Anti-Spike IgG; Middle left: Roche Anti-Nucleocapsid; Middle right: Roche Anti Spike/RBD; Bottom: GenScript neutralization surrogate test. Patients with lighter symptoms tend to have lower antibodies titre. The two groups do however not significantly differ, since the 95% confidence intervals overlap. **Figure S3** Bivariate comparisons shown as scatter plot for non-quantitative EI-S1-IgG vs quantitative EI-S1-IgG-quant. Dashed lines represent manufacturers’ cut-off values. The red solid line shows the LOESS (locally estimated scatterplot smoothing or local regression) estimation with CI. The black solid line with CI is a linear regression given for comparison. Square root R of coefficients of determination is given for association among continuous variables. Data presented in Rubio-Acero et al. [31].

## Data Availability

Data and code are accessible subject to data protection regulations upon reasonable request to the corresponding author.
