# Supplementary material for: Studying temporal titre evolution of commercial SARS-CoV-2 assays reveals significant shortcomings of using BAU standardization for comparison
Source: Virol J. 2023 Sep 1;20:200. doi: 10.1186/s12985-023-02167-z (PMC10474769; doi:10.1186/s12985-023-02167-z)
Supplement: Supplementary file 1 — Additional file 1: Figure S1 WHO grading of symptoms (from [29]). Figure S2 Longitudinal serology data of patients (PCR-positive cohort) over time colored by WHO symptom grade. Each line represents one subject, the dots represent the individual sample. All assays were performed from the same sample in a head-to-head comparison. The blue and red solid lines show the LOESS (locally estimated scatterplot smoothing or local regression) estimations with CI. Top left: Euroimmun Anti Spike IgA; Top right: Euroimmun Anti-Spike IgG; Middle left: Roche Anti-Nucleocapsid; Middle right: Roche Anti Spike/RBD; Bottom: GenScript neutralization surrogate test. Patients with lighter symptoms tend to have lower antibodies titre. The two groups do however not significantly differ, since the 95% confidence intervals overlap. Figure S3 Bivariate comparisons shown as scatter plot for non-quantitative EI-S1-IgG vs quantitative EI-S1-IgG-quant. Dashed lines represent manufacturers’ cut-off values. The red solid line shows the LOESS (locally estimated scatterplot smoothing or local regression) estimation with CI. The black solid line with CI is a linear regression given for comparison. Square root R of coefficients of determination is given for association among continuous variables. Data presented in Rubio-Acero et al. [31]. [file 12985_2023_2167_MOESM1_ESM.docx]

**Studying temporal titre evolution of commercial SARS-CoV-2 assays reveals significant shortcomings of using BAU standardization for comparison**

**Inge Kroidl^*,1,2^, Simon Winter^*,1^,** Raquel Rubio-Acero^1,3^, Abhishek Bakuli^1^, Christof Geldmacher^1,2^, Tabea M. Eser^1,2^, Flora Déak^1^, Sacha Horn^1^, Anna Zielke^1^, Mohamed I. M. Ahmed^1,2^, Paulina Diepers^1^, Jessica Guggenbühl^1^, Jonathan Frese^1^, Jan Bruger^1^, Kerstin Puchinger^1^, Jakob Reich^1^, Philine Falk^1^, Alisa Markgraf^1^, Heike Fensterseifer^1^, Ivana Paunovic^1,3,4^, Angelika Thomschke^1^, Michael Pritsch^1^, Friedrich Riess^1^, Elmar Saathoff^1^, Michael Hoelscher^1,2,4,5^, **Laura Olbrich^1,2✝^, Noemi Castelletti^1,4,6✝^, Andreas Wieser^1,2,3,4✝^**

on behalf of KoCo19/ORCHESTRA study group

^1^ Division of Infectious Diseases and Tropical Medicine, Medical Center of the University of Munich (LMU), Germany.

^2^ German Center for Infection Research (DZIF), partner site Munich, Germany.

^3^ Max-von-Pettenkofer Institute, LMU Munich, Germany

^4^ Fraunhofer Institute for Translational Medicine and Pharmacology ITMP, Immunology, Infection and Pandemic Research, Türkenstraße 87, 80799 Munich, Germany

^5^ Center for International Health (CIH), University Hospital, LMU Munich, 80336 Munich, Germany

^6^ Institute of Radiation Medicine, Helmholtz Zentrum M¨unchen, 85764 Neuherberg, Germany

* Share first authorship

✝ Share last authorship

**Keywords:** antibody; COVID-19; nucleocapsid; RBD; SARS-CoV-2; serology; spike; Binding Antibody Units

**Supplemental material:**

**Longitudinal serological dynamics after SARS-CoV-2 infection by WHO classification**

We determined the serological dynamics over time grouping the participants according to disease severity **(Supplemental Figure 2)**. The serological dynamics over time suggest a correlation between severity of symptoms and magnitude of serological response. However, there was no evidence for a statistical significance, as CIs overlapped. Lastly, we also explored grouping based on lower respiratory tract affection but did not observe any pattern (data not shown).

**Transformation of EI-S1-Ig into EI-S1-Ig-quant**

To compare the Ro-RBD-Ig assay with the EI-S1-Ig-quant assays we need both of them in the BAU unit, so that raw values can be directly compared. Therefore, all samples should be analyzed using the EI-S1-Ig-quant methods. Since most of the samples used for this manuscript were already emptied conducting the several assays presented, more analysis will not be possible. To overstep this issue we used paired EI-S1-Ig and EI-S1-Ig-quant samples already published [31]. The EI-S1-Ig-quant values can hence be extrapolated from EI-S1-Ig values using the LOESS (locally estimated scatterplot smoothing or local regression) function method modelling the association (**Supplemental Figure 3**) [31]. The LOESS estimation was preferred compared to a linear regression since it was modeling the data better at boundaries.

**Supplemental figures:**


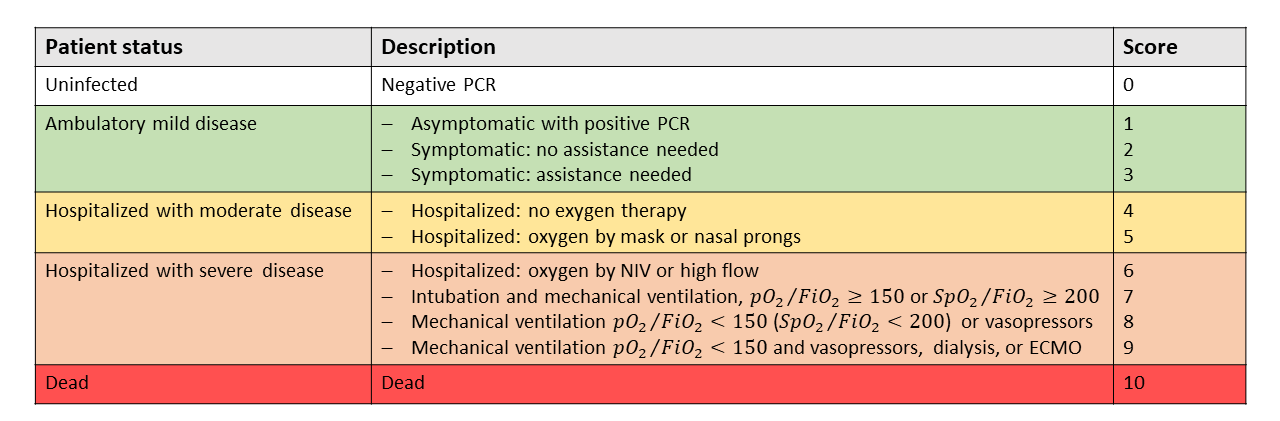


***Supplemental Figure 1:*** *WHO grading of symptoms (from [29])*


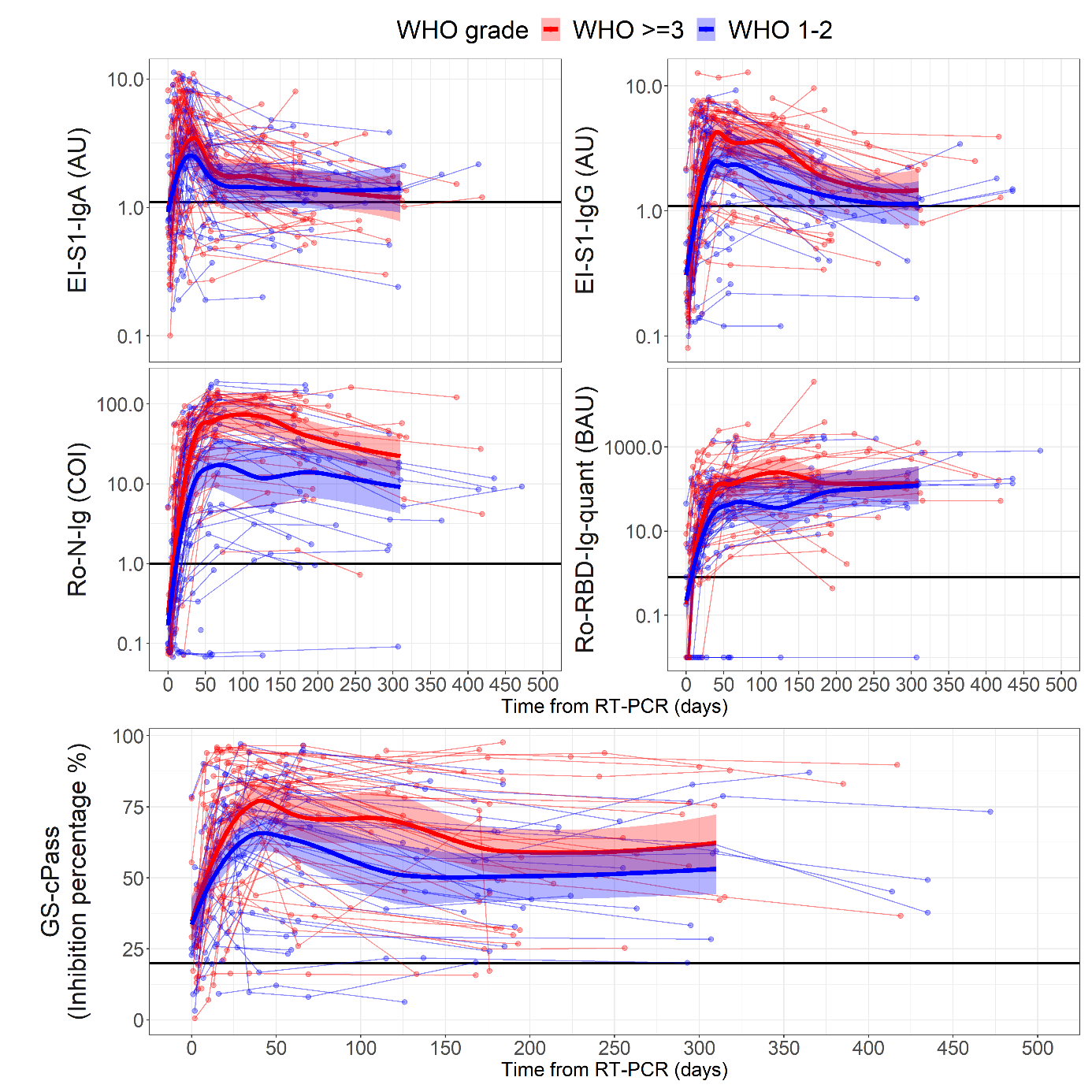


***Supplemental Figure 2:*** *Longitudinal serology data of patients (PCR-positive cohort) over time colored by WHO symptom grade. Each line represents one subject, the dots represent the individual sample. All assays were performed from the same sample in a head-to-head comparison. The blue and red solid lines show the LOESS (locally estimated scatterplot smoothing or local regression) estimations with CI. Top left: Euroimmun Anti Spike IgA; Top right: Euroimmun Anti-Spike IgG; Middle left: Roche Anti-Nucleocapsid; Middle right: Roche Anti Spike/RBD; Bottom: GenScript neutralization surrogate test. Patients with lighter symptoms tend to have lower antibodies titre. The two groups do however not significantly differ, since the 95% confidence intervals overlap.*


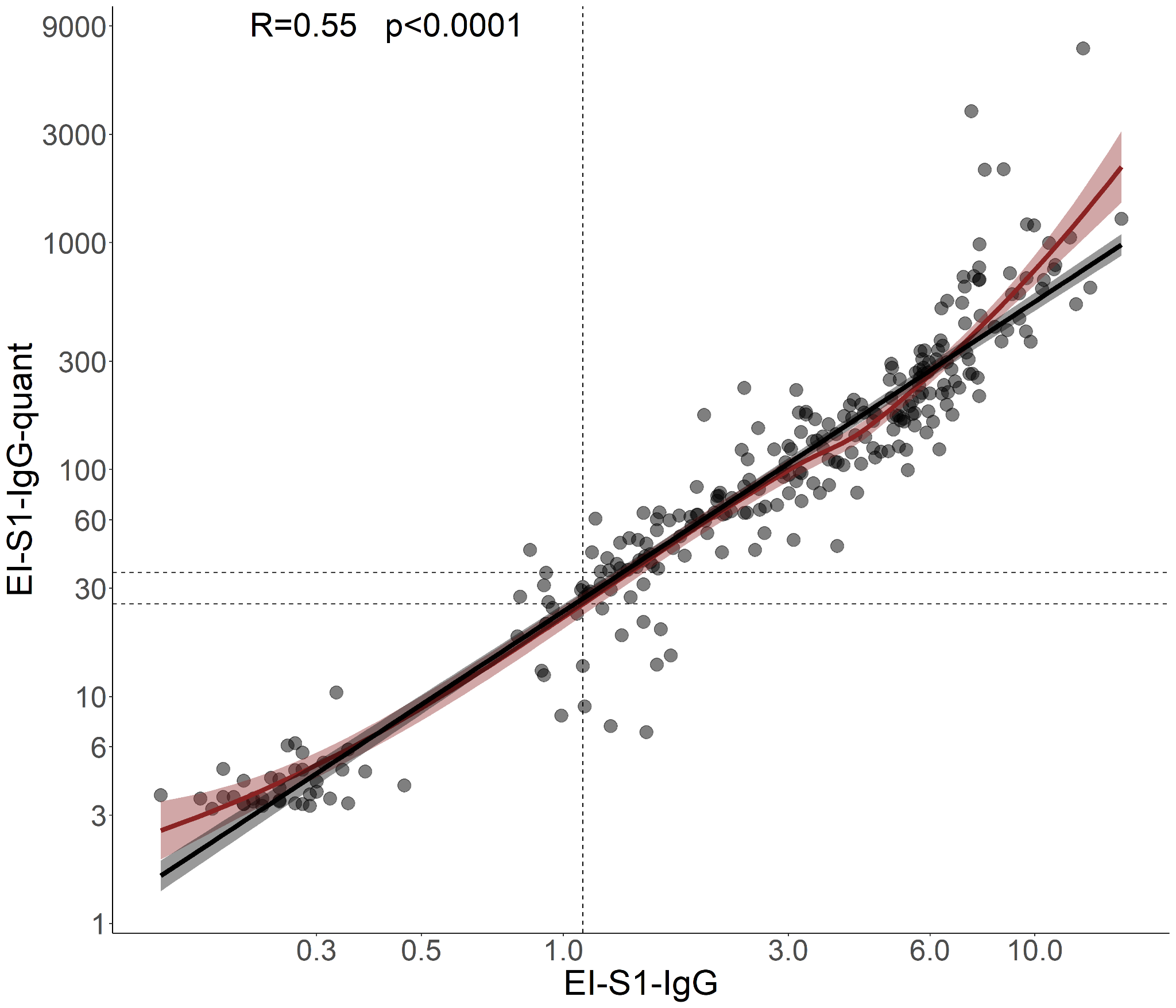


***Supplemental Figure 3:*** *Bivariate comparisons shown as scatter plot for non-quantitative EI-S1-IgG vs quantitative EI-S1-IgG-quant. Dashed lines represent manufacturers’ cut-off values. The red solid line shows the LOESS (locally estimated scatterplot smoothing or local regression) estimation with CI. The black solid line with CI is a linear regression given for comparison. Square root R of coefficients of determination is given for association among continuous variables. Data presented in Rubio-Acero, Castelletti [31].*

**References**

1. WHO. *Coronavirus disease (COVID-19) pandemic*. [01 July 2022]; Available from: https://www.who.int/emergencies/diseases/novel-coronavirus-2019.

2. CSSE. *Center for Systems Science and Engineering (CSSE) at Johns Hopkins University (JHU) COVID-19 Dashboard*. [01 July 2022]; Available from: https://coronavirus.jhu.edu/map.html.

3. Lumley, S.F., et al., *Antibody Status and Incidence of SARS-CoV-2 Infection in Health Care Workers.* N Engl J Med, 2021. **384**(6): p. 533-540.

4. Letizia, A.G., et al., *SARS-CoV-2 seropositivity and subsequent infection risk in healthy young adults: a prospective cohort study.* Lancet Respir Med, 2021. **9**(7): p. 712-720.

5. Polack, F.P., et al., *Safety and Efficacy of the BNT162b2 mRNA Covid-19 Vaccine.* N Engl J Med, 2020. **383**(27): p. 2603-2615.

6. Dagan, N., et al., *BNT162b2 mRNA Covid-19 Vaccine in a Nationwide Mass Vaccination Setting.* N Engl J Med, 2021. **384**(15): p. 1412-1423.

7. Baden, L.R., et al., *Efficacy and Safety of the mRNA-1273 SARS-CoV-2 Vaccine.* N Engl J Med, 2021. **384**(5): p. 403-416.

8. Voysey, M., et al., *Safety and efficacy of the ChAdOx1 nCoV-19 vaccine (AZD1222) against SARS-CoV-2: an interim analysis of four randomised controlled trials in Brazil, South Africa, and the UK.* Lancet, 2021. **397**(10269): p. 99-111.

9. Kim, J.H., F. Marks, and J.D. Clemens, *Looking beyond COVID-19 vaccine phase 3 trials.* Nat Med, 2021. **27**(2): p. 205-211.

10. Barros-Martins, J., et al., *Immune responses against SARS-CoV-2 variants after heterologous and homologous ChAdOx1 nCoV-19/BNT162b2 vaccination.* Nat Med, 2021. **27**(9): p. 1525-1529.

11. Caniels, T.G., et al., *Emerging SARS-CoV-2 variants of concern evade humoral immune responses from infection and vaccination.* Sci Adv, 2021. **7**(36): p. eabj5365.

12. Kroidl, I., et al., *Vaccine breakthrough infection and onward transmission of SARS-CoV-2 Beta (B.1.351) variant, Bavaria, Germany, February to March 2021.* Euro Surveill, 2021. **26**(30).

13. Hacisuleyman, E., et al., *Vaccine Breakthrough Infections with SARS-CoV-2 Variants.* N Engl J Med, 2021. **384**(23): p. 2212-2218.

14. Mizrahi, B., et al., *Correlation of SARS-CoV-2-breakthrough infections to time-from-vaccine.* Nat Commun, 2021. **12**(1): p. 6379.

15. Bergwerk, M., et al., *Covid-19 Breakthrough Infections in Vaccinated Health Care Workers.* N Engl J Med, 2021. **385**(16): p. 1474-1484.

16. Long, Q.X., et al., *Antibody responses to SARS-CoV-2 in patients with COVID-19.* Nat Med, 2020. **26**(6): p. 845-848.

17. Long, Q.X., et al., *Clinical and immunological assessment of asymptomatic SARS-CoV-2 infections.* Nat Med, 2020. **26**(8): p. 1200-1204.

18. Ibarrondo, F.J., et al., *Rapid Decay of Anti-SARS-CoV-2 Antibodies in Persons with Mild Covid-19.* N Engl J Med, 2020. **383**(11): p. 1085-1087.

19. Dan, J.M., et al., *Immunological memory to SARS-CoV-2 assessed for up to 8 months after infection.* Science, 2021. **371**(6529).

20. Flehmig, B., et al., *Persisting Neutralizing Activity to SARS-CoV-2 over Months in Sera of COVID-19 Patients.* Viruses, 2020. **12**(12).

21. Ripperger, T.J., et al., *Orthogonal SARS-CoV-2 Serological Assays Enable Surveillance of Low-Prevalence Communities and Reveal Durable Humoral Immunity.* Immunity, 2020. **53**(5): p. 925-933 e4.

22. Harris, R.J., et al., *Serological surveillance of SARS-CoV-2: Six-month trends and antibody response in a cohort of public health workers.* J Infect, 2021. **82**(5): p. 162-169.

23. Olbrich, L., et al., *Head-to-head evaluation of seven different seroassays including direct viral neutralisation in a representative cohort for SARS-CoV-2.* J Gen Virol, 2021. **102**(10).

24. Kristiansen, P.A., et al., *WHO International Standard for anti-SARS-CoV-2 immunoglobulin.* Lancet, 2021. **397**(10282): p. 1347-1348.

25. WHO. *WHO/BS.2020.2403 Establishment of the WHO International Standard and Reference Panel for anti-SARS-CoV-2 antibody*. [04 July 2022]; Available from: https://www.who.int/publications/m/item/WHO-BS-2020.2403.

26. AG, E.M.L. *Quantitativer Nachweis von Antikörpern gegen SARS-CoV-2*. 2021 [cited November 2022; Available from: https://www.coronavirus-diagnostik.de/documents/Indications/Infections/Coronavirus/EI_2606_I_DE_B.pdf.

27. Nordlab, L. *Testumstellung für den Nachweis von Anti-SARS-CoV-2 IgG*. Available from: https://www.nordlab.de/index.php/de/21-aktuelles/272-testumstellung-fuer-den-nachweis-von-anti-sars-cov-2-igg.

28. BV, M.D.B.; Available from: https://www.mediphos.be/en/news/who-standardized-igg-antibody-determination-against-whole-spike-sars-cov-2.

29. Characterisation, W.H.O.W.G.o.t.C. and C.-i. Management of, *A minimal common outcome measure set for COVID-19 clinical research.* Lancet Infect Dis, 2020. **20**(8): p. e192-e197.

30. Puchinger, K., et al., *The interplay of viral loads, clinical presentation, and serological responses in SARS-CoV-2 - Results from a prospective cohort of outpatient COVID-19 cases.* Virology, 2022. **569**: p. 37-43.

31. Rubio-Acero, R., et al., *In Search of the SARS-CoV-2 Protection Correlate: Head-to-Head Comparison of Two Quantitative S1 Assays in Pre-characterized Oligo-/Asymptomatic Patients.* Infect Dis Ther, 2021: p. 1-14.

32. Favresse, J., et al., *Persistence of Anti-SARS-CoV-2 Antibodies Depends on the Analytical Kit: A Report for Up to 10 Months after Infection.* Microorganisms, 2021. **9**(3).

33. Muecksch, F., et al., *Longitudinal Serological Analysis and Neutralizing Antibody Levels in Coronavirus Disease 2019 Convalescent Patients.* J Infect Dis, 2021. **223**(3): p. 389-398.

34. Chen, X., et al., *Disease severity dictates SARS-CoV-2-specific neutralizing antibody responses in COVID-19.* Signal Transduct Target Ther, 2020. **5**(1): p. 180.

35. Figueiredo-Campos, P., et al., *Seroprevalence of anti-SARS-CoV-2 antibodies in COVID-19 patients and healthy volunteers up to 6 months post disease onset.* Eur J Immunol, 2020. **50**(12): p. 2025-2040.

36. Seow, J., et al., *Longitudinal observation and decline of neutralizing antibody responses in the three months following SARS-CoV-2 infection in humans.* Nat Microbiol, 2020. **5**(12): p. 1598-1607.

37. Gudbjartsson, D.F., et al., *Humoral Immune Response to SARS-CoV-2 in Iceland.* N Engl J Med, 2020. **383**(18): p. 1724-1734.

38. Roltgen, K., et al., *Defining the features and duration of antibody responses to SARS-CoV-2 infection associated with disease severity and outcome.* Sci Immunol, 2020. **5**(54).

39. Krammer, F., *A correlate of protection for SARS-CoV-2 vaccines is urgently needed.* Nat Med, 2021. **27**(7): p. 1147-1148.

40. Feng, S., et al., *Correlates of protection against symptomatic and asymptomatic SARS-CoV-2 infection.* Nat Med, 2021. **27**(11): p. 2032-2040.

41. Haas, E.J., et al., *Impact and effectiveness of mRNA BNT162b2 vaccine against SARS-CoV-2 infections and COVID-19 cases, hospitalisations, and deaths following a nationwide vaccination campaign in Israel: an observational study using national surveillance data.* Lancet, 2021. **397**(10287): p. 1819-1829.

42. Shrotri, M., et al., *Spike-antibody waning after second dose of BNT162b2 or ChAdOx1.* Lancet, 2021. **398**(10298): p. 385-387.

43. Tang, P., et al., *BNT162b2 and mRNA-1273 COVID-19 vaccine effectiveness against the SARS-CoV-2 Delta variant in Qatar.* Nat Med, 2021. **27**(12): p. 2136-2143.

44. Levin, E.G., et al., *Waning Immune Humoral Response to BNT162b2 Covid-19 Vaccine over 6 Months.* N Engl J Med, 2021. **385**(24): p. e84.

45. Chemaitelly, H., et al., *Waning of BNT162b2 Vaccine Protection against SARS-CoV-2 Infection in Qatar.* N Engl J Med, 2021. **385**(24): p. e83.

46. Feikin, D.R., et al., *Duration of effectiveness of vaccines against SARS-CoV-2 infection and COVID-19 disease: results of a systematic review and meta-regression.* Lancet, 2022. **399**(10328): p. 924-944.

47. Scheiblauer, H., et al., *Antibody response to SARS-CoV-2 for more than one year - kinetics and persistence of detection are predominantly determined by avidity progression and test design.* J Clin Virol, 2022. **146**: p. 105052.
